# Alterations of the salivary microbiome in obstructive sleep apnea and their association with periodontitis

**DOI:** 10.3389/fcimb.2025.1642766

**Published:** 2025-09-09

**Authors:** Jiong Li, Jike Gao, Yunxia Ma, Wuli Li, Xiangru Chen, Zhenhua Li, Xiujun Zhang

**Affiliations:** ^1^ Key Lab. of Oral Diseases Research of Anhui Province, College & Hospital of Stomatology, Anhui Medical University, Hefei, China; ^2^ Department of Otorhinolaryngology, head and neck surgery, the first affiliated Hospital of Anhui Medical University, Hefei, China; ^3^ School of Mental Health and Psychological Sciences, Anhui Medical University, Hefei, China; ^4^ Department of Epidemiology and Biostatistics, School of Public Health, Anhui Medical University, Hefei, China

**Keywords:** obstructive sleep apnea, periodontitis, 16S rDNA, microbiome, Rothia

## Abstract

**Objective:**

Obstructive sleep apnea (OSA) and periodontitis have demonstrated epidemiological and clinical associations. This study aimed to characterize salivary microbiome alterations in patients with OSA, periodontitis, and their comorbidity (OSA+PD), and to explore potential microbial markers.

**Materials and methods:**

This cross-sectional study included 125 adults divided into four groups: healthy controls (H, n=26), patients with OSA (OSA/O, n=42), patients with periodontitis (PD/P, n=15), and patients with OSA and periodontitis (OSA+PD/OP, n=42). Participants underwent nocturnal polysomnography and comprehensive periodontal examinations. Saliva samples were collected and analyzed using 16S ribosomal DNA gene sequencing to evaluate microbial distribution and community structure across groups. Receiver operating characteristic (ROC) curves were generated for key taxa combining with clinical indicators, and the area under the curve (AUC) values were calculated to assess diagnostic relevance.

**Results:**

Oral microbial diversity was significantly altered in OSA, PD, and OSA+PD groups. Alpha diversity was reduced in all patient groups compared to healthy controls, with the periodontitis group showing the highest diversity and evenness. Beta diversity revealed that periodontitis having the strongest impact and the comorbid group exhibiting intermediate characteristics between OSA and periodontitis. Key taxa, including *Tannerella*, *Treponema*, *Prevotella*, *Slackia*, and *Streptococcus constellatus*, exhibited significant intergroup differences. BugBase phenotype analysis revealed an increased abundance of aerobic and a reduced presence of anaerobic microbial profiles in the OSA and OSA+PD groups. Additionally, *Rothia* and *Micrococcaceae* were more abundant in the OSA group, regardless of periodontal status. Receiver operating characteristic (ROC) analysis indicated that *Rothia* and *Parvimonas* reliably differentiated between OSA and OSA+PD (AUC=0.715, 0.702) and also between periodontitis and OSA+PD (*Rothia*: AUC=0.879).

**Conclusion:**

OSA is associated with distinct changes in salivary microbiota, including reduced microbial richness and altered functional profiles, which may contribute to early periodontal dysbiosis. *Rothia* has been identified as a potential microbial biomarker for OSA-related periodontitis, while *Rothia* and *Parvimonas* may play a key role in periodontitis-related OSA. However, as this is a cross-sectional study, causal relationships and the predictive value of microbial biomarkers remain to be confirmed in longitudinal studies. These results highlight the need for integrated management of OSA and periodontitis and suggest microbial profiling as a useful diagnostic tool.

## 1 Introduction

Obstructive sleep apnea (OSA) is the most prevalent sleep-related breathing disorder, affecting an estimated 936 million adults aged 30–69 years globally (Adam et al., 2019). Characterized by recurrent airway collapse during sleep, OSA leads to intermittent hypoxia and systemic oxidative stress, triggering chronic inflammation (Larissa et al., 2002; Suzanne et al., 2005; Lena and Peretz, 2009). This inflammatory state disrupts the microbial ecosystem. Studies have documented dysbiosis in the gut of OSA mouse models (Mohammad et al., 2022), and in the oral cavity (Chih-Yuan et al., 2019a) and upper respiratory tract (Xiaoman et al., 2023) of patients with OSA (Chih-Yuan et al., 2019b; Yi et al., 2023). Such microecological imbalances can further perpetuate inflammation (Valeriy et al., 2016; Aaron et al., 2020).

Periodontitis, a common inflammatory condition initiated by plaque biofilms, manifests as alveolar bone resorption, attachment loss, and periodontal pocket formation (Xiaoyu et al., 2021). It affects 61.6% of the global population (Diogo et al., 2023), with severe periodontitis ranking the sixth most prevalent disease, impacting over 1 billion individuals (Lubna and Ira, 2020; Gustavo et al., 2024). While research has advanced the understanding of the balance between plaque microbiota and periodontal immune responses, it remains unclear whether microbial dysbiosis is a cause or consequence of periodontitis (Michael et al., 2020). Moreover, periodontitis is closely linked to systemic conditions such as cardiovascular disease, diabetes, and elevated systemic inflammation (Sczepanik et al., 2020; Crystal et al., 2023; Koichiro et al., 2023; Maria Clotilde et al., 2023; Shinji et al., 2023).

OSA and periodontitis share multiple risk factors including age, male sex, smoking, alcohol use, diabetes, and obesity (David et al., 2023; Hiroyuki et al., 2023). Both conditions are associated with systemic diseases, suggesting a potential bidirectional interaction (Natalia Arango et al., 2023). While previous studies have reported a higher prevalence of periodontitis and elevated inflammatory markers in OSA patients (Hikmet et al., 2017; Mayra A et al., 2023; Seon-Rye et al., 2023; W-L et al., 2024), and case-control investigations have correlated OSA severity with periodontal parameters (e.g., probing depth, clinical attachment loss) and shifts in the subgingival microbiome (Nejat et al., 2015; Petra et al., 2021). Crucially, existing research on the salivary microbiome has predominantly focused on differences between OSA patients and healthy controls, or alterations induced by various interventions, while overlooking the significant impact of periodontitis status or excluding only severe periodontitis on salivary microbiota (Xuehui et al., 2022; Yujia et al., 2025). Thus, precise stratification according to periodontal health status is essential to unravel disease-specific microbial signatures and interaction mechanisms.

Saliva, as a non-invasively obtainable biological specimen (Anna et al., 2019), serves as a critical sample type for 16S rDNA sequencing analysis. It provides a unique non-invasive window for investigating the pathogenesis of periodontitis comorbidity (Lin et al., 2020). while simultaneously exhibiting responsive changes to OSA-related pathophysiological alterations (e.g., flow rate, viscosity, and pH variations) (Nicolò et al., 2022; Jiyoung et al., 2023; Supanigar et al., 2023). Furthermore, compared to subgingival plaque sampling, saliva collection demonstrates significantly reduced technical complexity and operator dependency.

Therefore, this study uniquely addresses this gap by conducting a comprehensive analysis of the salivary microbiota across four meticulously defined cohorts: healthy controls (H), periodontitis-only patients (P), OSA-only patients (O), and patients with both conditions (OP) using 16S rDNA gene sequencing. The objectives included identifying changes in periodontal microbial complexes, determining significant differential bacteria among groups, assessing the predictive value of specific bacteria for these diseases, and correlating microbiota profiles with clinical indicators of both periodontitis and OSA. Considering that existing studies have focused on the effects of OSA and PD on salivary microbiota, our analysis emphasizes the effect of periodontitis on salivary microbiota (O vs. OP) in OSA patients, and the effect of OSA on salivary microbiota (P vs. OP) in patients with periodontitis.

## 2 Method

### 2.1 Study participants

A total of 125 participants were recruited from the Stomatological Hospital of Anhui Medical University and the First Affiliated Hospital of Anhui Medical University in Anhui, China. Participants were divided into four groups based on clinical indicators of OSA and periodontitis: (i) 15 patients with periodontitis (Group P), (ii) 42 patients with OSA syndrome (OSAS) (Group O), (iii) 42 patients with comorbid OSA and periodontitis (Group OP), and (iv) 26 healthy individuals without periodontitis, OSA, or systemic diseases (Group H). This study was approved by the Ethics Committee of the Affiliated Stomatological Hospital of Anhui Medical University (approval no. T2023015) and adhered to the Declaration of Helsinki.

Inclusion Criteria: age between 18 and 70 years, provision of informed consent, presence of ≥20 remaining teeth meeting basic oral function, absence of contraindications for periodontal examination.

Exclusion Criteria: use of antibiotics within the past 3 months, periodontal therapy within the past 6 months, smoker, history of pharmacological or surgical treatment for OSA, treatment with continuous positive airway pressure (CPAP) or bilevel positive airway pressure (BPAP), pregnancy or breastfeeding, presence of acute illnesses (e.g., cold, pharyngitis, urinary tract infections, gastroenteritis), systemic diseases (e.g., cardiovascular, diabetes, pulmonary, hepatic, neurological conditions), and infectious diseases (e.g., syphilis, AIDS).

### 2.2 Diagnosis of OSA

OSA was diagnosed based on polysomnographic monitoring (PSG) in Snoring Center, Department of Otorhinolaryngology - Head and Neck Surgery, The First Affiliated Hospital of Anhui Medical University, an apnea-hypopnea index (AHI) threshold and lower oxygen saturation (LSaO_2_). AHI is calculated as the total number of apneas and hypopneas per hour of sleep. In adults, the presence of OSA was determined as an AHI score of ≥5, while mild OSA is defined as an AHI of at least 5 to 15 events per hour, moderate OSA as >15 to 30 events per hour, and severe OSA as >30 events per hour (Michael, 2014). 84 participants were classified as having OSA, while 41 did not.

### 2.3 Assessment of clinical periodontal indicators

Periodontitis was diagnosed and classified according to the 2018 International Classification of Periodontitis (Tonetti) (Maurizio S et al., 2018). Clinical periodontal examinations were conducted by an experienced dentist (Y.C.) using index teeth to represent whole-mouth periodontal conditions. Periodontal parameters such as probing pocket depth (PPD), clinical attachment loss (CAL), and plaque index (PI), were measured at six sites per tooth using a Williams-type periodontal probe (Thin Williams Probe POW6; Hu-Friedy, Inc.). Measurements were rounded off to the nearest millimeter. Periodontal examinations further categorized the participants into 57 individuals with periodontitis and 68 without.

### 2.4 Saliva sample collection

Saliva samples were collected in the morning, either after PSG or on the same day as the periodontal examination. Participants rinsed their mouths with 10 ml of water for 30 seconds and expectorated before collection. Unstimulated whole-saliva samples were collected using the Navazesh method (Mahvash, 1993). Participants were instructed to refrain from oral hygiene activities (e.g., brushing, rinsing, or gargling), eating, drinking, or chewing gum for at least one hour before collection. A minimum of 2 ml of unstimulated saliva was collected into a sterile test tube and immediately frozen at -80 °C for subsequent analysis.

### 2.5 DNA extraction

Genomic DNA was extracted from archived saliva samples using Tianamp Bacteria DNA Kit (Tiangen biochemical technology (Beijing) Co.; Ltd.; Beijing; China) following the manufacturer’s protocol. The V3-V4 hypervariable region of the bacterial 16S rDNA gene was amplified using modified primers 341F (5’-CCTACGGNGGCWGCAG-3’) and 805R (5’-GACTACHVGGGTATCTAATCC-3’).

### 2.6 16S rDNA gene sequencing and dataset construction

Sequencing was performed on an Illumina NovaSeq platform according to the manufacturer’s standard guidelines. Paired-end reads were assigned to samples based on unique barcodes, and barcodes and primers were trimmed. A total of 10,521,693 raw reads were generated from the 125 saliva samples. Reads were merged using FLASH, and raw reads were quality-filtered using fqtrim (v0.94). Chimeric sequences were removed using VSearch (v2.3.4), 65,172 high-quality reads were identified, resulting in 1,076 operational taxonomic units (OTUs) shared between healthy and diseased groups. De-replication was conducted using DADA2, generating feature tables and sequences. These were used to create Amplicon Sequence Variant (ASV) feature lists for diversity, taxonomic annotation, and differential abundance analyses (Mark et al., 2005). This process yielded ASV feature tables and 10,171 feature sequences for all 125 patient samples, forming a comprehensive dataset for downstream analysis.

### 2.7 Statistical and sequencing data analysis

Age, weight, and BMI were assessed for variability among groups using the Kruskal-Wallis test, and cohens’f was used to evaluate the effects of these differences on the results. Continuous variables with a normal distribution were expressed as mean ± standard deviation and analyzed using the Student’s t-test or ANOVA. Non-normally distributed variables are reported as median and interquartile range (25th–75th percentile) and analyzed using the Kruskal-Wallis test. Categorical variables were presented as frequencies and percentages and analyzed using the chi-square test. To assess the robustness of our statistical analyses, a *post hoc* power analysis was conducted using G*Power software (version 3.1.9.7).

Alpha diversity was assessed using the Chao-1, Observed OTUs, Goods coverage, Shannon, Simpson indices and Pielou_evenness. Beta diversity was evaluated using two distance metrics, unweighted UniFrac and Jaccard, and analyzed through Nonmetric Multidimensional Scaling (NMDS), permutational multivariate analysis of variance (Adonis), and analysis of similarities (ANOSIM). Significant differences in bacterial abundance between groups were identified using the LEfSe method. Heat maps were normalized with Z-scores to standardize bacterial abundance values.

To explore relationships between bacterial abundance and clinical factors (e.g., age, weight, and BMI), linear regression was applied to adjust for potential confounders. Functional category predictions were performed using PICRUSt2 (Gavin et al., 2020), and statistical analyses and visualizations were conducted using STAMP. Redundancy analysis (RDA) and Spearman’s correlation were used to investigate microbial variations in relation to clinical indicators of periodontitis and OSA. Additionally, receiver operating characteristic (ROC) curves were generated for key taxa, and the area under the curve (AUC) values were calculated to assess diagnostic relevance.

All statistical analyses were conducted using IBM SPSS Statistics (SPSS Inc.), and a P value of < 0.05 was considered statistically significant.

## 3 Results

### 3.1 Characteristics of study subjects

A total of 125 participants were included in the study, with their baseline characteristics summarized in **Table 1**. Participants with OSA (Groups O and OP) demonstrated significantly compared to the periodontitis-only (P) and healthy control (H) groups (*P* < 0.001).

**Table 1:** Characteristics of study participants.

Characteristics	Health	OSA	OSA+PD	PD	P value	Cohen’s f
n	26	42	42	15		
Age, yr	35.115 (18-58)	39.024 (23-60)	47.238 (27-68)	39.267 (23-59)	<0.001	0.074
Sex, female/male	15/11	35/7	39/3	7/8		
Height (cm)	169.115 (154-183)	170.952 (153-195)	169.69 (152-181)	165.533 (150-183)	0.191	0.049
Weight (Kg)	68.712 (44-95)	80.129 (55-110)	82.271 (56-120)	74.767 (60-106)	<0.001	0.072
BMI (Kg/m2)	23.842 (17.6-32.9)	27.398 (20.6-36.5)	28.624 (19,4-38.7)	27.42 (21.7-38.9)	<0.001	0.075
Clinical indicators of obstructive sleep apnea						
AHI (times/h)	2.046 (0-4.5)	39.448 (5.2-85.7)	58.286 (30.3-95.8)	1.247 (0-4.9)	<0.001	0.16
LSaO2 (%)	88.423 (78-95)	71.548 (38-88)	63.214 (30-89)	89.333 (76-95)	<0.001	0.142
Periodontal clinical indicators						
PPD (mm)	1.843 (1.25-2.47)	1.831 (1.14-2.69)	5.457 (4.75-6.31)	5.48 (4.67-7.28)	<0.001	0.179
CAL (mm)	0 (0,0)	0 (0,0)	3.555 (2.95-4.31)	3.573 (2.87-5.18)	<0.001	0.181
PI	1.635 (1,3.17)	1.758 (1,3.17)	3.27 (2,4,75)	2.789 (2,3.67)	<0.001	0.181

OSA, obstructive sleep apnea; PD, periodontitis; BMI, body mass index; AHI, apnea-hypopnea index; LSaO2, lower oxygen saturation; PPD, probing depth; CAL, clinical attachment loss; PI, plaque index.

Part of values are expressed as Average value (maximum-minimum).

Age, weight, and BMI were significantly different among groups. However, the effect sizes for these differences were minimal (Cohen’s f: 0.074–0.075) and aligned with known epidemiological patterns, such as the higher prevalence of OSA among older males with elevated BMI (Tianyi et al., 2018; Caterina and Giovanna, 2021). Multiple linear regression was applied to adjust for these variables (Benjamin et al., 2019), identifying bacteria associated with age, weight, and BMI for exclusion from subsequent analyses (*P* < 0.05).

In addition, based on the sample sizes of the four groups (P: 15, O: 42, OP: 42, H: 26) and the observed intergroup differences in PPD. Given a significance level of α = 0.05, the statistical power (1 − β) was calculated to exceed 0.99, supporting the robustness of our findings despite the relatively small sample size of Group P.

### 3.2 Alpha diversity analysis

**Figure 1:**
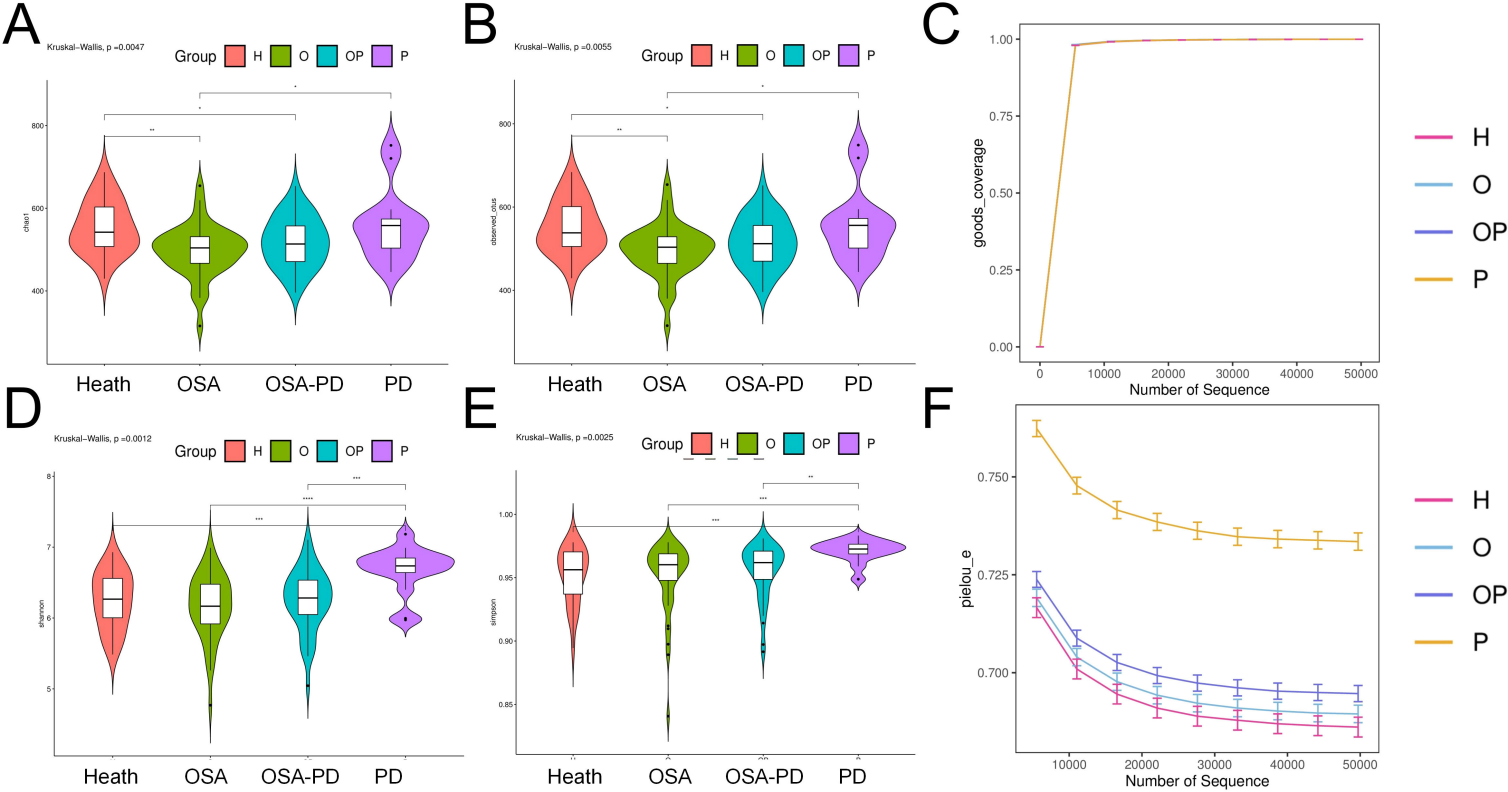
Alpha diversity of salivary microbiota across study groups. **(A)** Chao1 Index; **(B)** Observed OTUs; **(C)** Goods Coverage; **(D)** Shannon Index; **(E)** Simpson Index; **(F)** Pielou’s Evenness. The Chao1 index and observed OTUs indicate significant differences between groups. Statistical significance: *P < 0.05, **P < 0.01, ***P < 0.001, ****P < 0.0001. (H, Healthy control; O, Obstructive sleep apnea; OP, Obstructive sleep apnea with periodontitis; P, Periodontitis).

OSA and periodontitis reshape oral microbial communities through different pathological mechanisms, while comorbidities show unique microbial “intermediate” characteristics. Taxonomic classification identified 2,332 phyla, 4,479 classes, 9,961 orders, 15,947 families, 26,515 genera, and 33,196 species across all samples. Species richness of the microbial communities was assessed using the Chao1 index and Observed OTUs index. The results demonstrated a significant reduction in species richness in both OSA, O and OP groups compared to the healthy control group (**Figures 1A, B**). The Goods coverage index approached 1 across all experimental groups, indicating adequate sequencing depth that effectively captured the microbial diversity within the samples, ensuring the representativeness of the results (**Figure 1C**). Representing the combined measure of species richness and evenness, the Shannon and Simpson indices revealed that the P group exhibited the highest alpha diversity, while the OP group displayed a relatively lower level of diversity (**Figures 1D, E**). Analysis of the Pielou evenness index (Pielou e) showed that species distribution evenness was consistently high (Pielou e ≈ 0.70) in H, O, and OP groups. In contrast, the P group demonstrated slightly higher evenness (Pielou e ≈ 0.75) (**Figure 1F**).

### 3.3 Beta diversity analysis

**Figure 2:**
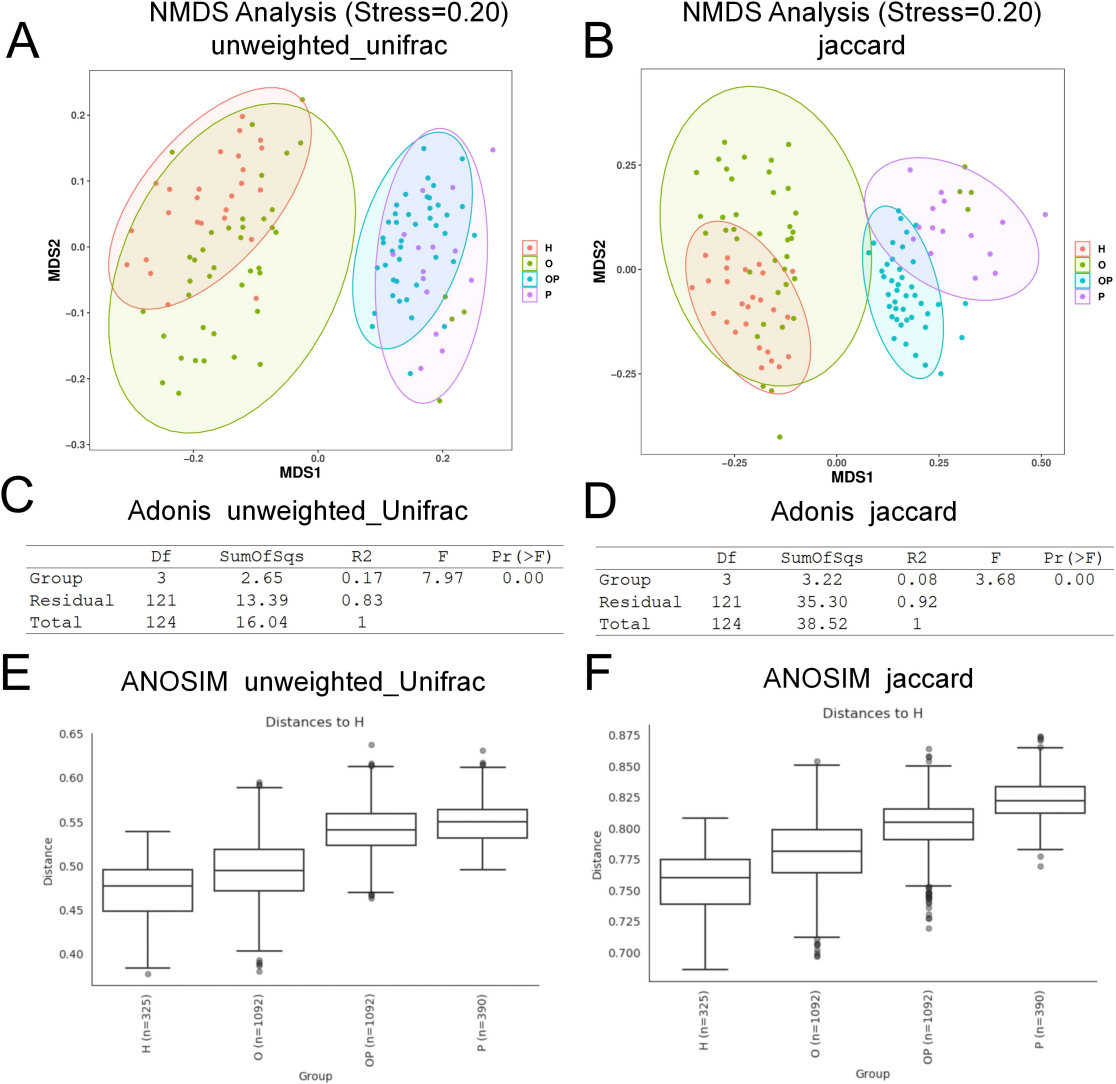
Beta diversity analysis of salivary microbial communities. **(A)** Unweighted NMDS; **(B)** Jaccard_NMDS; **(C)** Unweighted UniFrac Adonis analysis; **(D)** Jaccard Adonis analysis; **(E)** Unweighted UniFrac anosim clustering; **(F)** Jaccard ANOSIM clustering. The results demonstrate clear group differences in microbial community structure and diversity.

Beta diversity analyses using unweighted UniFrac and Jaccard distance metrics revealed that periodontitis exerted the greatest impact on microbial community differences across groups. NMDS ordination, which relies on the rank order of dissimilarity coefficients (reflecting the relative order of samples rather than actual distances), revealed a gradual increase in microbial community dissimilarity from the H group to the O/OP and P groups (**Figures 2A, B**). Both distance metrics also demonstrated that the OSA and P groups had a high explanatory power for sample classification in Adonis (unweighted UniFrac: R² = 0.83; Jaccard: R² = 0.92) (**Figures 2C, D**). Further analysis using ANOSIM confirmed that the differences in microbial composition between the patient groups (O, OP, P) and the healthy control group (H) progressively increased (**Figures 2E, F**).

### 3.4 Microbial distribution and periodontal complex differences

**Figure 3:**
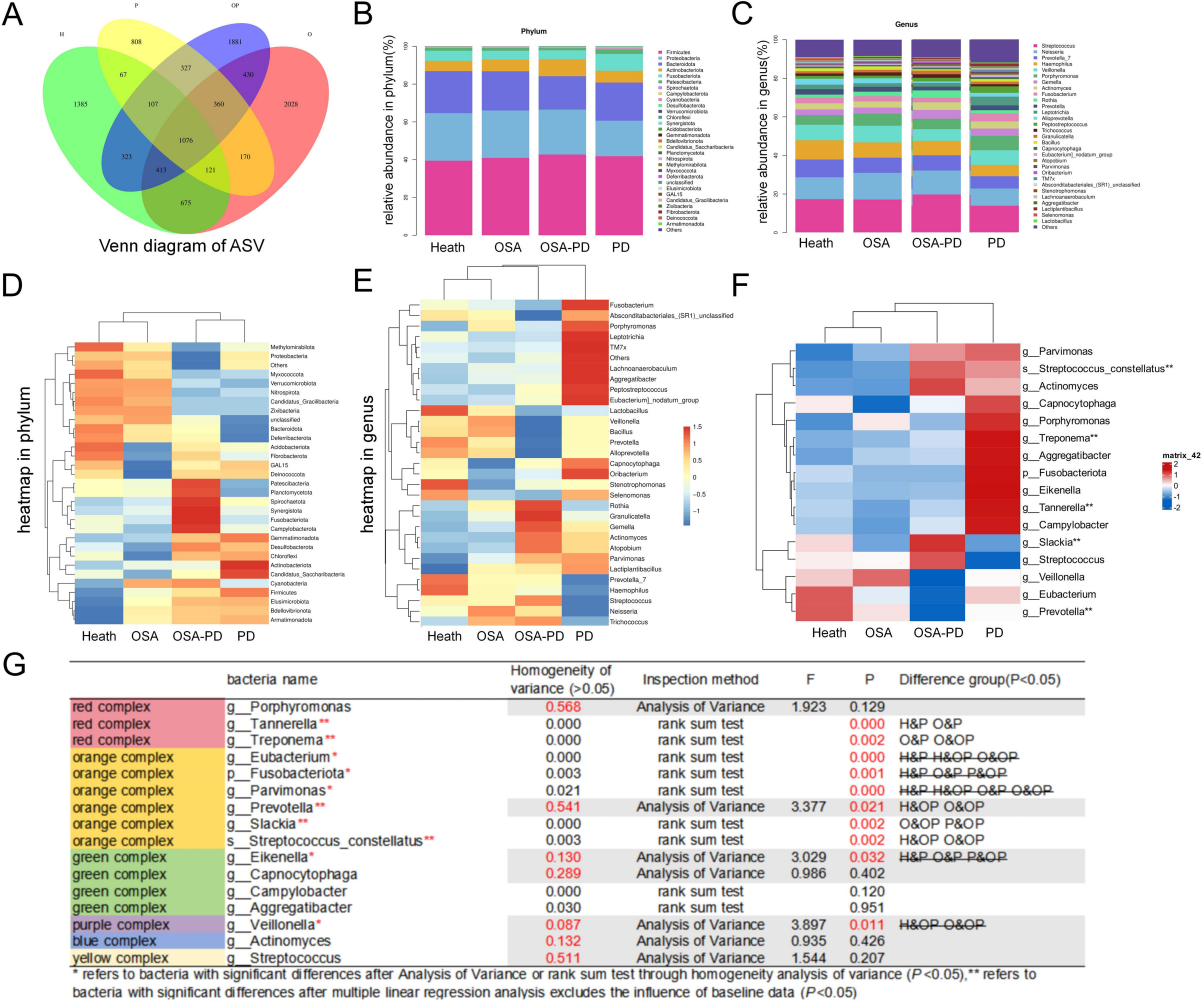
Microbial composition and structure at the genus level. **(A)** Venn diagram showing shared and unique ASVs among groups; **(B)** Relative abundance of the top 30 bacterial taxa at the phylum level across groups; **(C)** Relative abundance of the top 30 bacterial taxa at the genus level across groups; **(D)** Heatmap of the top 30 bacterial taxa at the phylum level; **(E)** Heatmap of the top 30 bacterial taxa at the genus level; **(F)** Heatmap showing the abundance of periodontal complexes across groups; **(G)** Differential analysis of periodontal complexes between groups. *P < 0.05; **indicates significant intergroup differences in bacterial abundance after adjusting for baseline data using multivariate linear regression.

A Venn diagram of ASV features (**Figure 3A**) highlighted the number of unique ASVs in each group: healthy (H, 1,385), OSA (2,028), OSA with periodontitis (1,881), and periodontitis only (808). Total ASVs were highest in the OSA group (5,273), followed by OSA with periodontitis (4,917), healthy controls (4,167), and periodontitis-only (3,036). The 30 most abundant taxa were analyzed at the phylum, genus, and OTU levels (**Figures 3B, C**). Heatmaps of taxonomic composition (**Figures 3D, E**) provided visual comparisons of bacterial distributions across groups.

Key periodontitis-associated bacteria were examined to assess their influence on microbial communities in different disease states. The analysis focused on Socransky’s periodontal complexes (Socransky et al., 1998), which classify subgingival bacteria into six groups based on their aggregation patterns and pathogenicity. Differential bacteria identified across the groups included *Tannerella*, *Treponema, Prevotella, Slackia*, and *Streptococcus constellatus* (**Figure 3G**). Ten periodontal complex species were ranked among the top 30 taxa at phylum, genus, and class levels. A heatmap of the abundance of these bacteria is shown in **Figure 3F**, excluding the confounding effects of weight, BMI, and age.

### 3.5 Bacterial distribution and function

**Figure 4:**
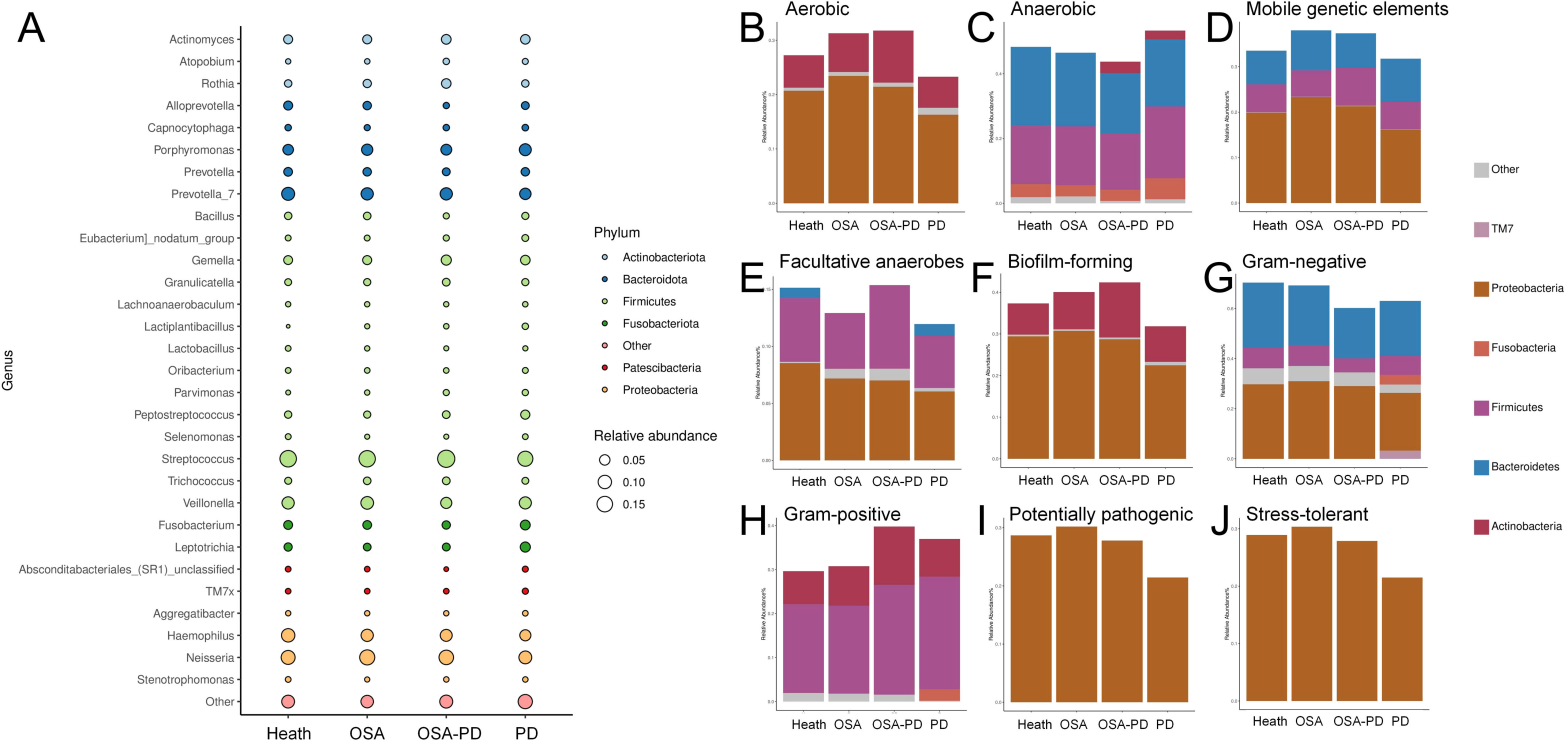
Top five bacteria and functional phenotypes. **(A)** Relationships between phylum and genus; **(B)** Aerobic bacteria; **(C)** Anaerobic bacteria; **(D)** Bacteria with mobile genetic elements; **(E)** Facultative anaerobes; **(F)** Biofilm-forming bacteria; **(G)** Gram-negative bacteria; **(H)** Gram-positive bacteria; **(I)** Potentially pathogenic bacteria; **(J)** Stress-tolerant bacteria.

The distribution of bacterial phyla and genera across groups is shown in the bubble diagram (**Figure 4A**). Dominant phyla included *Bacteroidota*, *Firmicutes*, and *Proteobacteria*. Functional phenotypes were analyzed using BugBase, which characterizes the relationship between phyla and bacterial functions. Consistent with previous findings, the periodontitis group displayed fewer aerobic and more anaerobic bacterial phenotypes, along with an increase in non-adherent plaques and a higher prevalence of both Gram-negative and Gram-positive bacteria. Conversely, the OSA (O and OP groups) displayed a richer aerobic bacterial phenotype and fewer anaerobic phenotypes, and exhibited a higher prevalence of mobile genetic elements and increased biofilm-forming capabilities (**Figures 4B–J**).

### 3.6 Prediction of metabolic pathways in differential bacteria

**Figure 5:**
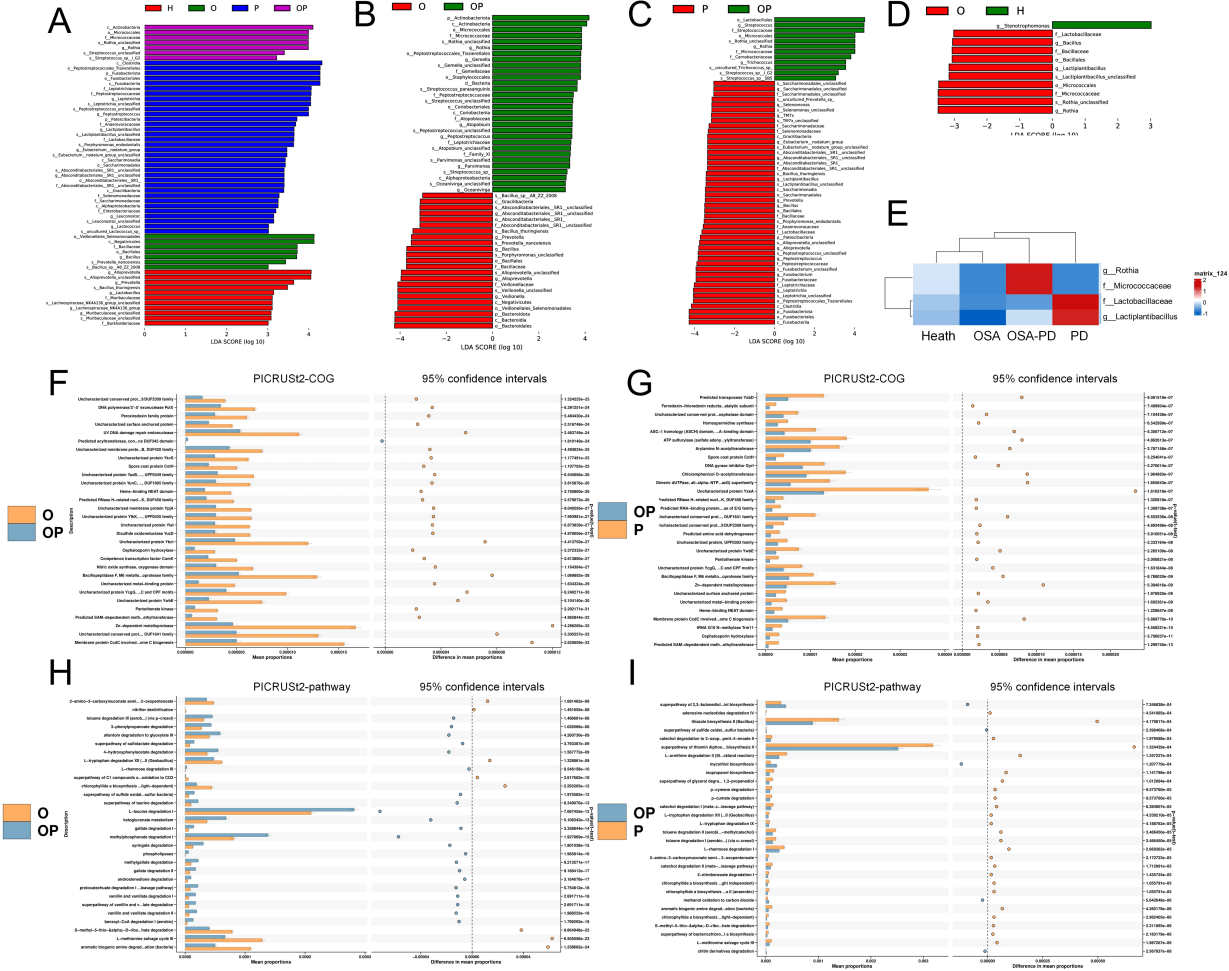
Differential bacteria and predicted metabolic pathways. **(A)** Differential bacteria across H, O, OP, and P groups; **(B)** Differential bacteria between O and OP groups; **(C)** Differential bacteria between P and OP groups; **(D)** Differential bacteria between O and H groups; **(E)** Comparison of differential bacteria in OSA (O/OP) versus non-OSA (H/P) groups. **(F, G)** PICRUSt2-COG and STAMP analyses of metabolic pathway differences in O vs. OP and P vs. OP groups, respectively; **(H, I)** Detailed pathway analysis using PICRUSt2-pathway and STAMP for O vs. OP and P vs. OP groups, identifying key metabolic shifts.

Key bacterial taxa with significantly differing abundances between groups were identified using LEfSe analysis. These biomarkers are summarized in **Figure 5A**. Specific comparisons were conducted to understand the effect of periodontitis and osa on saliva microorganisms. Effect of PD on OSA saliva microorganisms (O vs. OP Groups): *Rothia*, *Gemella*, and *Oceanivirga* were significantly enriched in the OP group (**Figure 5B**). Effect of OSA on PD saliva microorganisms (P vs. OP groups): *Streptococcus* and *Rothia* were notably enriched in the OP group (**Figure 5C**).

Analysis of OSA’s impact on the oral microbiome revealed that *Rothia* and *Micrococcaceae* were abundant in the OSA groups (OSA and OSA+PD). In contrast, in the non-OSA groups (H and PD), *Lactobacillaceae* and *Lactiplantibacillus* were more prevalent in healthy controls (H) than in patients with OSA. This trend reversed among individuals with PD (**Figure 5E**).

### 3.7 Functional predictions of oral microbiota

The metabolic functions of the oral microbiota were predicted using PICRUSt2. STAMP analysis identified 16 shared metabolic pathways of OP. Effect of PD on OSA saliva microorganisms (O vs. OP Groups): In the OP group, L-leucine degradation I was significantly less expressed, while pathways such as L-methionine salvage cycle III and aromatic biogenic amine degradation were less expressed (**Figure 5F**). Effect of OSA on PD saliva microorganisms (P vs. OP groups): In the OP group, pathways such as ATP sulfurylase [involved in sulfate assimilation (Laura et al., 2014)] and Arylamine N-acetyltransferase [a conjugating enzyme linked to acetyl-coenzyme A metabolism (Edith et al., 2008)] were significantly enriched (**Figure 5G**).

### 3.8 Differentiating potential of major bacterial taxa for OSA with periodontitis comorbidity

**Figure 6:**
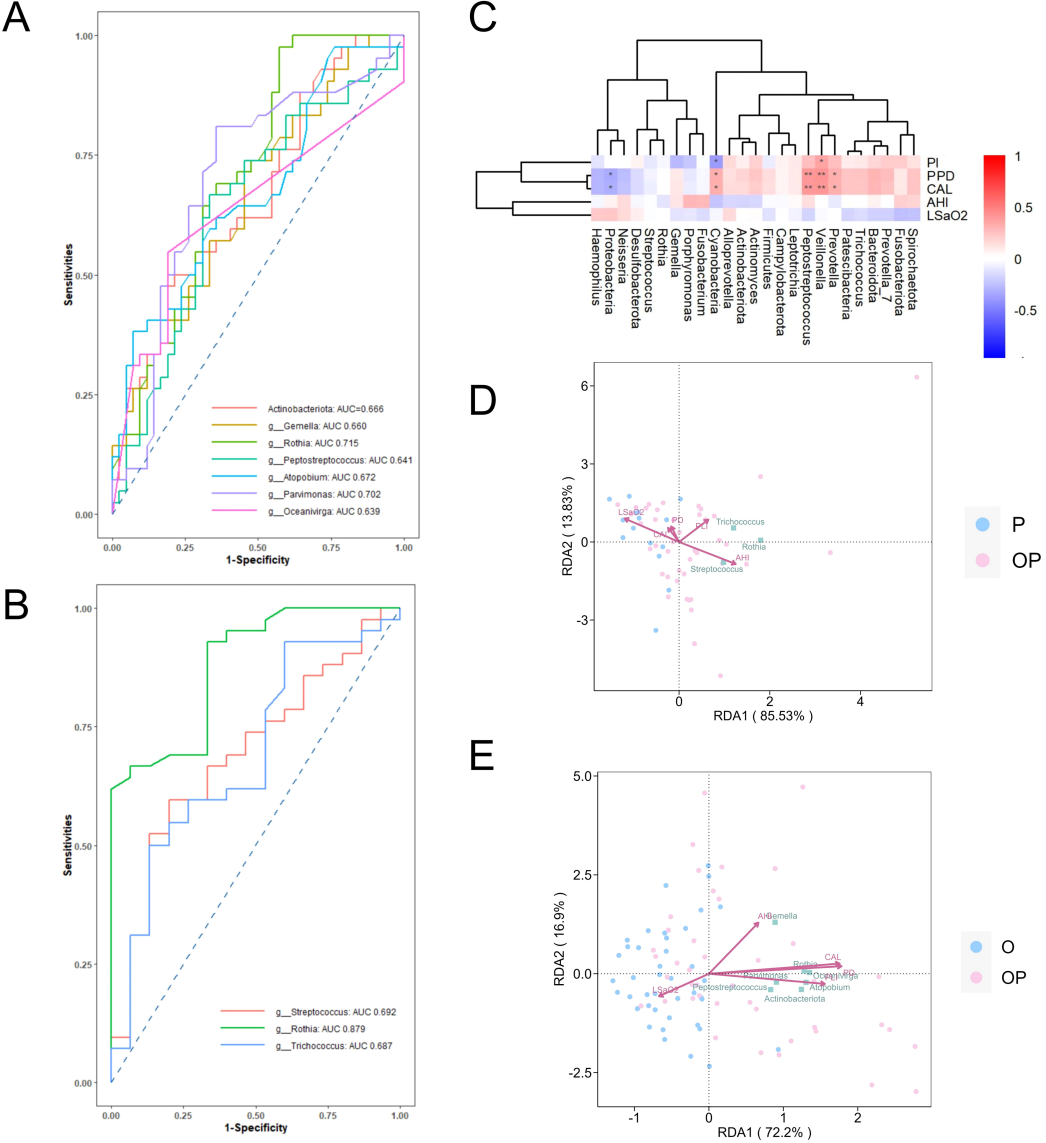
Predictive value of differential bacteria and correlations with clinical indicators. **(A)** ROC curves for the prediction of periodontitis in O patients transitioning to OP; **(B)** ROC curves for the prediction of OSA in P patients transitioning to OP; **(C)** Spearman correlation analysis showing the relationships between the top 10 phyla and top 15 genera in the OP group with periodontal (CAL, PPD, PI) and sleep disorder (AHI, LSaO2) clinical indicators; **(D)** RDA analysis of differential bacteria (phylum and genus levels) in the O-OP group, correlating with periodontal (PPD, PI, CAL and sleep apnea (AHI, LSaO2) indicators; **(E)** RDA analysis of differential bacteria (genus level) in the P-OP group, correlating with periodontal (CAL, PPD, PI) and sleep apnea (AHI, LSaO_2_) indicators.

The differentiating capability of bacterial taxa for OSA with periodontitis (OP) comorbidities was assessed using ROC curves (**Figures 6A, B**). Differential taxa identified in the O-OP and P-OP groups included *Actinobacteriota*, *Rothia*, *Gemella*, *Atopobium*, *Peptostreptococcus*, *Parvimonas*, *Oceanivirga*, *Streptococcus*, and *Trichococcus*.

For differences between the O and OP, *Rothia* and *Parvimonas* demonstrated moderate differentiating accuracy, with area under the curve (AUC) values of 0.715 and 0.702, respectively. Other taxa, including Actinobacteriota, *Gemella*, *Atopobium*, *Peptostreptococcus*, and *Oceanivirga*, showed lower differentiating performance (AUC range: 0.639–0.672). For differences between the P and OP, *Rothia* exhibited high differentiating accuracy (AUC = 0.879), while *Streptococcus* and *Trichococcus* showed moderate differentiating power (AUC = 0.692 and 0.687, respectively).

### 3.9 Correlation between microbial variations and clinical indicators

The relationship between microbial diversity and clinical indicators was examined using Spearman’s correlation analysis, with baseline data included as covariates (**Figures 6A–C**). At the phylum level, cyanobacteria positively correlated with PPD (r = 0.32, P = 0.041), and CAL (r = 0.31, P = 0.043), while negatively correlating with PI (r = -0.38, P = 0.013). Proteobacteria negatively correlated with PPD (r = -0.33, P = 0.031) and CAL (r = -0.31, P = 0.043). At the genus level, *Veillonella*, *Peptostreptococcus*, and *Prevotella* showed positive correlations with PPD (r = 0.42, P = 0.006; r = 0.39, P = 0.010; r = 0.32, P = 0.039, respectively) and CAL (r = 0.40, P = 0.009; r = 0.40, P = 0.010; r = 0.31, P = 0.044, respectively), while *Veillonella* also correlated positively with PI (r = 0.35, P = 0.022).

To further investigate the interplay between periodontitis and OSA, the O-OP and P-OP groups were analyzed using RDA and LEfSe. Differential bacteria were identified at the phylum and genus levels, and their associations with clinical indicators of PD (CAL, PPD, and PI) and OSA (AHI and LSaO_2_) were characterized (**Figures 6D, E**).

## 4 Discussion

The association between obstructive sleep apnea (OSA) and periodontitis in the context of oral microbiology has yielded inconsistent results in previous studies (Peizeng et al., 2021; Xiaoman et al., 2021; Yanlong et al., 2021; Yu et al., 2022). For instance, Jia et al. and Chen et al. both reported no significant differences in salivary microbiome alpha diversity between OSA and non-OSA groups. However, Chen et al. noted lower microbial abundance in the OSA group. Notably, neither study stratified participants based on periodontal status, which may have confounded their results. Building on these findings, our study demonstrates that OSA, regardless of periodontitis status, is associated with reduced microbial abundance. Both α- and β-diversity analyses indicated that OSA profoundly impacts the salivary microbial composition of periodontitis. Further exploration of the different bacterial taxa confirmed that OSA affected the abundance of key periodontal pathogens. Interestingly, patients with OSA exhibited subclinical microbial imbalances that may contribute to early periodontal inflammation, differing from the more pronounced dysbiosis observed in PD-only individuals. Comparisons between the microbial profiles of the OSA and OSA+PD groups, and the PD and OSA+PD groups revealed significant differences in bacterial species and metabolic functions. ROC analysis further identified *Rothia* as a potential biomarker for predicting the transition from OSA or PD to OSA+PD, underscoring its predictive value. Incorporating clinical indicators also highlighted correlations between bacterial species and both periodontal and sleep-related clinical measures.

OSA is characterized by repeated upper airway obstruction during sleep, leading to fragmented sleep, intermittent hypoxia, and systemic inflammation. Alpha diversity analysis indicated a reduction in microbial richness in OSA patients (**Figures 1A, B**), along with attenuated microbial diversity in OSA+PD compared to PD alone (**Figure 1D**). Pielou’s evenness remained consistent across Health, OSA, and OSA+PD groups (~0.7; **Figure 1E**), the overall lower diversity in OSA and OSA+PD suggests a microbial community in an early successional or degraded state compared with PD. Our functional analyses (**Figures 4B–J**) revealed significant shifts in bacterial traits, including altered aerobic to anaerobic and Gram-positive to Gram-negative bacterial ratios. These shifts may result from increased mouth breathing and altered oral ventilation in patients with OSA, which influence environmental factors like oxygen levels and humidity.

In the β-diversity analysis, the oral microbial community structure in the OSA (O) and OSA+PD (OP) groups was more similar to the healthy controls (H) than to the periodontitis-only group (P) (**Figures 2E, F**). This observation suggests two possible interpretations: (1) individuals with OSA may develop periodontal inflammation before significant dysbiosis occurs; or (2) the microbial diversity in PD patients is suppressed by OSA-associated alterations, such as reduced species richness. However, previous studies have reported no significant association between periodontitis and OSA in patients with class III obesity (Sílvia Helena de Carvalho et al., 2016), lending more support to the first hypothesis—that OSA may facilitate the onset of periodontal inflammation even in the absence of advanced microbial imbalance.

To better interpret the β-diversity patterns, we considered potential explanations for the observed microbial clustering. OSA and PD independently change the composition of oral microbial organisms: OSA patients are characterized by aerobic bacteria enrichment (such as *Streptococcus*, *Micrococciaceae*) and enhanced biofilm formation ability, while PD patients are mainly imbalanced anaerobic bacterial groups dominated by periodontal pathogenic bacteria (such as *Treponema*, *Prevotella*). Complications (OSA+PD) present unique “intermediate” microbial characteristics, and their community structure is closer to the healthy group, suggesting that the oxidative stress of OSA may partially offset the anaerobic environment of the periodontal pocket and inhibit the absolute advantage of periodontal pathogens.

Interestingly, studies on the upper airway mucosa in patients with OSA have not identified significant differences in microecological environments compared to non-OSA individuals (Seung-No et al., 2021). Similarly, conditions like asthma–which affect ventilation–show minimal associations with periodontitis (Ana et al., 2023; Tamiya et al., 2023). However, the severity of OSA has been shown to correlate with elevated nitric oxide (NO) concentrations in exhaled breath (Dang-Thi-Mai et al., 2021), which may also influence periodontal inflammation (Lima et al., 2024). Altered NO metabolism may underlie the link between OSA and periodontitis. Our data show that nitrifier denitrification and NO synthase pathways were enriched in OSA compared to OSA+PD (Holger et al., 2016; Raquel Mantuaneli et al., 2017), indicating a shift in oral nitrogen cycling (**Figures 5F–I**). Previous studies have reported that the periodontal microbiome in periodontitis exhibits reduced nitrate- and nitrite-reducing capacity (Bob et al., 2024), contributing to impaired NO homeostasis. This dysregulation may help explain the observed association between OSA severity and periodontitis stage (Zhiqiang et al., 2022). These findings suggest that future research should consider microbial NO metabolism as a potential mechanistic link between the two conditions. Despite this, the impact of ventilation status on oral microbial profiles in OSA remains underexplored and warrants further investigation.

Periodontitis is a well-established disease that is associated with oxidative stress. Meanwhile periodontitis is initiated by plaque biofilms, characterized by a shift from supragingival to subgingival plaques at the macroscopic level and from Gram-positive to Gram-negative bacterial dominance. Our findings (**Figures 4G, H**) reflect this “transitional phenomenon,” though shift in the OSA+PD group was less pronounced than in the P group, suggesting that hypoxemia and hypercapnia in patients with OSA may increase periodontal tissue susceptibility to inflammation. Mechanisms such as the modulation of innate immunity, alterations in the osteoblast-osteoclast balance, and oxygen-related signaling pathway changes may play a role. Reports indicate that Intermittent hypoxia and subsequent reoxygenation enhance alveolar bone resorption in human periodontal ligament cells (Motohira et al., 2007). Studies using periodontitis rat models have shown that chronic intermittent hypoxia impairs periodontal bone formation by downregulating osteogenic markers such as RUNX2 and MDM21 (Li et al., 2016). Hypoxia-inducible factor-1α (HIF-1α), an oxygen-dependent transcription activator, is a key regulator of periodontal tissue and alveolar bone metabolism. Bacterial-induced HIF-1α activation–arising from inflammation or immune responses–may exacerbate the condition (Pearl et al., 2020). Oxidative stress induced by OSA provides a plausible explanation for the increased severity of periodontitis in patients with comorbid OSA and PD (Mayra A et al., 2022). These findings highlight the importance of hypoxia-related mechanisms in the pathogenesis of periodontitis and its exacerbation in patients with OSA.

Using LEfSe analysis to identify strains with significant variability between groups, we observed distinct shifts in the oral microbiota during the transition from a single condition (OSA or periodontitis) to a combined comorbidity of OSA and periodontitis (OSA+PD group). At the phylum level, *Gemella* was significantly enriched in the patients with OSA+PD. As a commensal periodontal bacterium with opportunistic pathogenic potential (Julián et al., 2023), *Gemella* has been associated with atherosclerotic plaques and endocarditis (Abarna et al., 2020). Given its association with cardiovascular infections, the enrichment of Gemella in OSA+PD raises the hypothesis of increased cardiovascular risk (Eberhard et al., 2016), which warrants further epidemiological investigations. Notably, both OSA and periodontitis patients transitioning to OSA+PD showed significant enrichment of *Rothia* in saliva. The potential use of *Rothia* as a biomarker is underscored by its predictive value for disease progression (Osamu et al., 2017).

Further analysis using ROC curves revealed that *Rothia* and *Parvimonas* demonstrated acceptable accuracy in predicting the transition of patients with OSA to OSA with periodontitis comorbidity (OSA+PD) (AUC = 0.715 and 0.702, respectively). Additionally, *Rothia* showed high predictive accuracy for determining whether periodontitis progressed to OSA+PD (AUC = 0.879). **Figure 5E** also shows that *Rothia* was an independent bacterium related to health vs. OSA and PD vs. OSA+PD. Meanwhile, *Parvimonas*, a member of the periodontal orange complex, may influence subgingival dysbiosis by enhancing the expression of periodontal virulence factors, potentially affecting treatment outcomes. On the other hand, *Rothia*, a Gram-positive opportunistic pathogen, is known for its infectious potential, particularly in immunocompromised individuals. *Rothia*, along with the differential genus *Prevotella*, has also been implicated as an etiological agent of halitosis, suggesting that respiratory patterns may influence bacterial abundance within the periodontal microbiome.

Correlation analysis elucidated the relationship between bacterial abundance and clinical indicators in patients with OP. For example, *Veillonella* was positively correlated with periodontal indicators, such as PPD and PI, while *Cyanobacteria* showed a significant negative correlation with PI. Interestingly, clinical indicators of sleep disorders were not significantly correlated with oral microbiota, indicating that periodontal status has a more pronounced influence on the oral microbiota composition in patients with OSA+PD. This finding aligns with our β-diversity analyses, which demonstrated that periodontal conditions exert greater ecological effects than sleep disorders on the oral microbiome. Statistical analysis of differentially expressed bacteria in the OSA and OSA/OSA+PD groups, combined with RDA, showed that obstructive apnea severity and oral hygiene significantly influenced the microbial differences between periodontitis to OSA+PD. However, the inflammatory changes in the periodontal tissues were less pronounced. Among the taxa analyzed, *Atopobium* exhibited a strong positive correlation with PPD, PI, CAL, and AHI, but was negatively correlated with LSaO2. As an anaerobic Gram-positive bacterium primarily studied in the context of vaginal inflammation (Andrea et al., 2019), *Atopobium* may also play a role in the oral microbiome under hypoxic and inflammation (Kıvılcım et al., 2023).

Overall, these findings suggest that targeting respiratory conditions such as OSA may indirectly influence changes in oral microbial composition, particularly affecting taxa such as *Rothia* and *Atopobium*. Future research should further explore these relationships to elucidate the potential mechanisms linking OSA, periodontitis, and systemic health outcomes.

Patients with comorbid OSA and periodontitis often present with more severe periodontal disease. To mitigate baseline differences in clinical periodontal indicators between the PD and OSA+PD groups, we increased the sample size. Nonetheless, as a cross-sectional study, our design limits the ability to infer temporal or causal relationships, particularly in identifying predictive microbial biomarkers. In addition, this study had several other limitations. Therefore, we regret that it is not clear what effect OSA and PD will have on the microbiota characteristics of patients with complications when they are the first diseases respectively, and we could not track microbiota changes during the progression of OSA and periodontitis. Secondly, the use of saliva samples limits the representation of periodontal plaques. Finally, we focus exclusively on the bacterial microbiota via 16S rDNA gene sequencing. As a result, fungal communities (e.g., *Candida* spp.) may have a more obvious imbalance in periodontitis (Kimil et al., 2022; Yao et al., 2024), which requires 18S rRNA or ITS sequencing for detection, but were not evaluated. Future research could address these limitations by employing longitudinal study designs, increasing OSA severity classification, incorporating lower respiratory tract sampling, improving species-level microbial analysis, and paying comprehensive attention to all kinds of microorganisms to explore how treatment for periodontal disease and OSA affects the oral microbiota.

## 5 Conclusion

This study demonstrates that periodontitis, OSA, and their comorbidity (OSA+PD) significantly reshape the composition and function of the salivary microbiome. OSA appears to predispose individuals to periodontal inflammation even before overt dysbiosis occurs, potentially serving as an early risk factor for periodontitis. The identification of microbial biomarkers such as Rothia and their association with clinical parameters highlights their potential for predicting disease progression. ROC analyses further support the diagnostic value of Rothia in identifying patients at risk of developing comorbid OSA and periodontitis.

These findings underscore the clinical importance of integrated approaches to managing OSA and periodontal disease. In particular, addressing sleep-disordered breathing during periodontal treatment may help restore oral microbial balance and prevent disease progression in patients with comorbid conditions. Future longitudinal studies are warranted to validate these associations and to explore the effects of therapeutic interventions on the oral microbiome in this population. We also recommend that future salivary microbiome studies on periodontitis take OSA status into account during sample collection and analysis to minimize potential confounding effects.

## Data Availability

The data presented in the study are deposited in the National Center for Biotechnology Information repository, accession number ID 1292140.
